# A Kinetically Controlled Bioconjugation Method for the Synthesis of Radioimmunoconjugates and the Development of a Domain Mapping MS-Workflow for Its Characterization

**DOI:** 10.1021/acs.bioconjchem.3c00519

**Published:** 2024-02-17

**Authors:** Marco A. Pometti, Giuseppe Di Natale, Giancarlo Geremia, Nileshgiri Gauswami, Gianni Garufi, Giuseppina Ricciardi, Marcella Sciortino, Fabrizio Scopelliti, Giorgio Russo, Massimo Ippolito

**Affiliations:** † Nuclear Medicine Department, Cannizzaro Hospital, Via Messina 829, 95126 Catania, Italy; ‡ FORA S.p.A., Via Alfred Bernhard Nobel 11/a, 43122 Parma, Italy; § CNR-Istituto di Cristallografia, Via Paolo Gaifami 18, 95126 Catania, Italy; ∥ Parco scientifico e tecnologico della Sicilia S.C.P.A., Stradale Vincenzo Lancia 57, 95121 Catania, Italy; ⊥ IBFM-CNR Institute of Molecular Bioimaging and Physiology, Contrada Pietra Pollastra, 90015 Cefalù, Italy

## Abstract

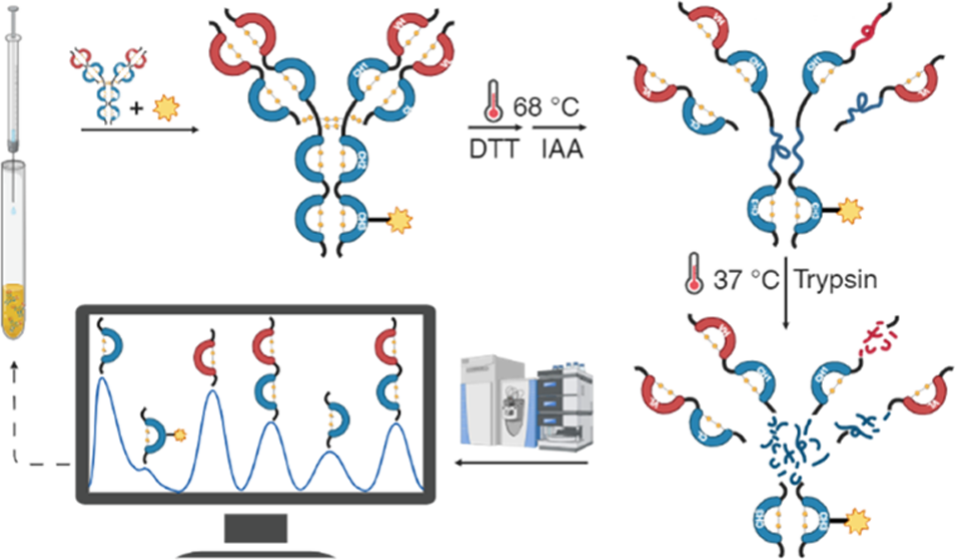

Immunoconjugates exploit the high affinity of monoclonal antibodies for a recognized antigen to selectively deliver a cytotoxic payload, such as drugs or radioactive nuclides, at the site of disease. Despite numerous techniques have been recently developed for site-selective bioconjugations of protein structures, reaction of ε-amine group of lysine residues with electrophilic reactants, such as activated esters (NHS), is the main method reported in the literature as it maintains proteins in their native conformation. Since antibodies hold a high number of lysine residues, a heterogeneous mixture of conjugates will be generated, which can result in decreased target affinity. Here, we report an intradomain regioselective bioconjugation between the monoclonal antibody Trastuzumab and the *N*-hydroxysuccinimide ester of the chelator 2,2′,2″,2‴-(1,4,7,10-tetraazacyclododecane-1,4,7,10-tetrayl)tetraacetic acid (DOTA) by a kinetically controlled reaction adding substoichiometric quantities of the activated ester to the mAb working at slightly basic pH. Liquid chromatography–mass spectrometry (LC–MS) analyses were carried out to assess the chelator-antibody ratio (CAR) and the number of chelating moieties linked to the mAb chains. Proteolysis experiments showed four lysine residues mainly involved in bioconjugation (K188 for the light chain and K30, K293, and K417 for the heavy chain), each of which was located in a different domain. Since the displayed intradomain regioselectivity, a domain mapping MS-workflow, based on a selective domain denaturation, was developed to quantify the percentage of chelator linked to each mAb domain. The resulting immunoconjugate mixture showed an average CAR of 0.9. About a third of the heavy chains were found as monoconjugated, whereas conjugation of the chelator in the light chain was negligible. Domain mapping showed the CH3 domain bearing 13% of conjugated DOTA, followed by CH2 and VH respectively bearing 12.5 and 11% of bonded chelator. Bioconjugation was not found in the CH1 domain, whereas for the light chain, only the CL domain was conjugated (6%). Data analysis based on LC–MS quantification of different analytical levels (intact, reduced chains, and domains) provided the immunoconjugate formulation. A mixture of immunoconjugates restricted to 15 species was obtained, and the percentage of each one within the mixture was calculated. In particular, species bearing 1 DOTA with a relative abundance ranging from 4 to 20-fold, in comparison to species bearing 2DOTA, were observed. Pairing of bioconjugation under kinetic control with the developed domain mapping MS-workflow could raise the standard of chemical quality for immunoconjugates obtained with commercially available reactants.

## Introduction

The advent of so-called biological therapies, especially based on monoclonal antibodies (mAbs), deeply changed the management of several cancers.^1,2^ To date, more than a hundred mAbs have been FDA and EMA approved, of which nearly half (42.6%) for cancer treatment.^3^ Nevertheless, these drugs still show limitations, mainly due to the emergence of mechanisms of resistance^4^ which hinder the long-term survival of cancer patients.^5^ Moreover, in the case of solid tumors, limited tissue penetration, mainly due to mAbs large size, affects the overall efficiency of treatment.^6^ Thus, research is moving toward improvements of these drugs. One of the most traveled roads in this direction is the creation of immunoconjugates, with the aim of enhancing the cytotoxic action of mAbs;^7^ in particular, antibody-drug conjugates (ADCs) are the most developed. A specific subclass of immunoconjugates are the radioimmunoconjugates, which exploits the high affinity of mAbs for a recognized antigen to selectively deliver a radioactive nuclide, allowing for diagnostic and therapeutic nuclear medicine applications.^8^ Radioimmunoconjugates can be synthesized by covalently binding the radionuclide to amino acid residues of mAbs (direct strategy), as in the case of iodine radionuclides bonded to tyrosine residues, or by covalently binding a chelator moiety to amino acid residues of mAbs, able to complex the radionuclide (indirect strategy), which is the case object of this study. The compounds synthesized by an indirect strategy have shown a better tumor to background ratio due to the residualizing nature of radiocatabolites generated after cellular internalization.^9^ To date, only two radioimmunoconjugates, [^90^Y]ibritumomab tiuxetan (Zevalin) and [^131^I]tositumomab (Bexxar), both full-length and used in radioimmunotherapy (RIT) of non-Hodgkin’s lymphoma (NHL), reached FDA approval,^10^ of which the latter retired for market reasons.^8^ No mAb-based nuclear imaging probes have yet reached the FDA or EMA approval. The reluctance to approve mAb-based radiopharmaceuticals by regulatory agencies is reasonably explainable with the high radiation dose received from patients because of the high physical half-lives of radionuclides used to match the biological half-lives of mAbs (about 21 days for IgG1). Despite these drawbacks, there is a plenty of scientific literature of clinical studies employing full-length and mAb-fragments derived radioimmunoconjugates both for imaging^11^ and therapy^12^ because of their great potential.

An aspect of fundamental importance in the creation of radioimmunoconjugates is to preserve the affinity of the mAb for the recognized antigen after the bioconjugation reaction, which is obtained by modifying the mAb as low as possible, especially in the complementarity-determining regions (CDRs).^13^ Several site-selective bioconjugation methods have been implemented, most of all working on non-native forms of mAb: genetically engineered mAbs (cysteine point mutations), chemically modified glycosylation sites of mAbs, using enzymatic post-translational modification and including unnatural amino acids in the polypeptide backbone.^14^ The few site-selective methods working on native mAbs are computationally assisted and use tailored reactants,^15,16^ which is a perspective not easily implementable in a nuclear medicine facility. Thus, the most used modification methods for the creation of radioimmunoconjugates involve the use of reactive electrophilic groups such as activated esters, isothiocyanate (SCN),^17^ isocyanate, and anhydrides of the chelator which reacts with the ε-amino group of lysine residues.^18^ Most of the reported methods in the literature use *N*-hydroxysuccinimide (NHS) esters of the chelator in large excess (chelator/Ab ratio ranging from 5:1 to more than 100:1) with pH ranging from 8.5 to 9.5.^19−21^ When such a type of procedure is employed, heterogeneous mixtures of the native mAb and the corresponding modified mAb are obtained, bearing different numbers of chelators for antibody randomly bonded to different sites, mainly lysine residues exposed to the solvent, with loss in immunoreactivity as the chelator-antibody ratio (CAR) increases.^22^ Moreover, NHS esters exhibit a typical half-life of just 10 min at pH 8.6 and 4 °C,^23^ preventing work at constant stoichiometric ratios.^24^ Actually, it is well-known that it is possible to modify the ε-amino group of lysine in proteins working at slightly basic pH because of the different chemical environment to which each residue is exposed in the three-dimensional (3D) conformation,^25^ pH values at which the half-life of NHS esters increases to 4–5 h.^26^ In 2012, Chen et al. obtained with low yields a site-selective biotinylation of smaller protein structures ribonuclease A (RNase A), lysozyme C, and peptide SST-14 using biotin-LC-NHS ester at a pH value of 7.2 in a kinetically controlled (KC) reaction.^27^

It is important to note that at pH values lower than those traditionally used in mAb modification, only a limited number of lysine residues contain the deprotonated amino group able to react as nucleophilic reactants. Only these lysine residues will be able to react, with a higher rate, with electrophilic reactants added in substoichiometric quantities over time, obtaining a mixture of immunoconjugates restricted to a few species. Starting from these assumptions, we considered the reaction between Trastuzumab, a humanized IgG1 mAb used for immunotherapy of human epidermal growth factor receptor 2 (HER2) positive tumors,^28^ and the *N*-hydroxysuccinimide ester of the chelator 2,2′,2″,2‴-(1,4,7,10-tetraazacyclododecane-1,4,7,10-tetrayl)tetraacetic acid (DOTA), one of the most used chelator for theragnostic applications.^29^ The kinetic control was achieved using an infusion pump (see Figure S1 in SI). The bioconjugation reaction was also carried out without kinetic control (one-step bioconjugation) using the same experimental conditions.

Bioconjugations were monitored through liquid chromatography–mass spectrometry (LC–MS) analyses. The resulting immunoconjugates DOTA-Trastuzumab were characterized in intact mass mode to assess the chelator-antibody ratio and in middle-up mode to fix the number of chelator moieties linked to mAb chains. Furthermore, proteolysis experiments were conducted to assess residues involved in kinetically controlled bioconjugation. In particular, the percentage of chelator linked to each mAb domain was assessed, developing a domain mapping MS-workflow in which the immunoconjugate was denatured by heating during reduction and alkylation steps. In these reaction conditions, selective domain unfolding allowed collection of the remaining folded domains after trypsin digestion. Finally, data analysis based on LC–MS quantification of different analytical levels (intact, reduced chains, and domains) provided a molecular formulation of the mixture of immunoconjugates.

## Results and Discussion

The possibility of pairing a single radioactive nuclide with a mAb molecule, as in the case of radiopharmaceuticals based on small molecules, is an attractive prospect, especially for diagnostic purposes. In the case of indirect radiolabeling of mAbs, the aforementioned possibility can be obtained by synthesizing immunoconjugates having unitary CAR. The use of commercially available reactants, such as NHS esters, requires the involvement of a limited number of lysine residues, especially not affecting the CDR functions, in order to preserve the affinity of mAb for the antigen. This implies the need for an analytical technique capable of evaluating the efficiency of the conjugation reaction and provides clear indications capable of improving the experimental procedure.

**Figure 1:**
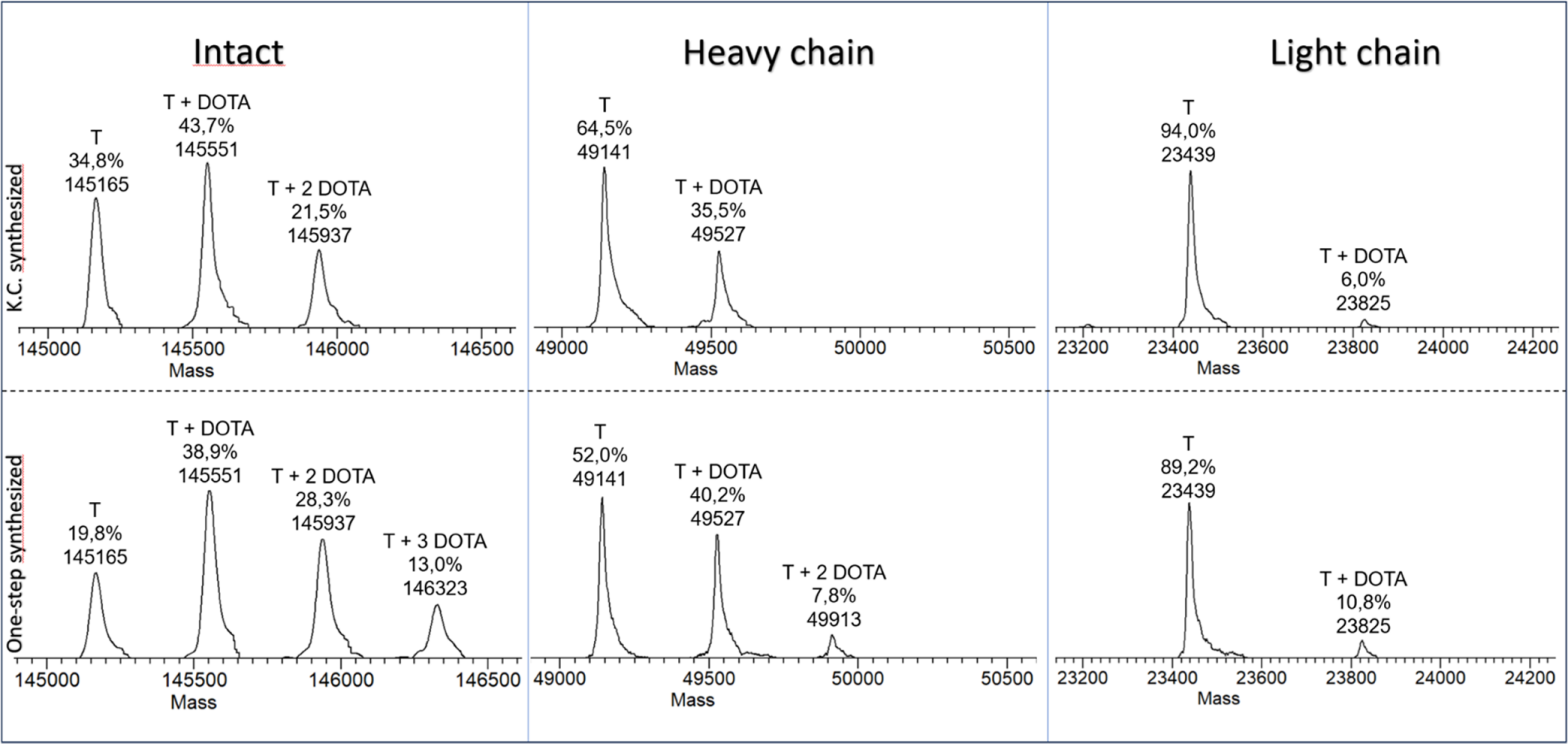
Deconvoluted electrospray ionization mass spectrometry (ESI-MS) spectra of deglycosilated immunoconjugates (KC up and one-step synthesized down): intact mass analysis (on the left), deglycosilated heavy chain (in the middle), and light chain (on the right). *T* = Trastuzumab.

Trastuzumab contains 88 lysins of which more than one-third are highly solvent exposed and 4 N-terminal groups,^30^ which could be modified through conjugation reaction. When the bioconjugation is performed using the protocols generally used in the literature (namely carbonate buffer at pH 9.2; mostly overnight),^20,31^ half of the ε-amino groups of lysine residues are deprotonated, and the half-life of DOTA-NHS is less than 10 min at 4 °C. In these reaction conditions, LC–MS analysis showed a complete conjugation reaction after a few minutes, and a fast and extensively randomized conjugation could occur. Therefore, we resorted to a kinetically controlled (KC) bioconjugation in order to reduce the number of lysine residues involved in the reaction. In particular, we added 0.01 equiv per minute of DOTA-NHS to Trastuzumab at room temperature and slightly basic pH, monitoring the degree of modification through LC–MS analysis. The reaction was stopped at a DOTA-NHS/Trastuzumab ratio of 5:1. The same reaction conditions were also applied in the one-step approach for comparison (see Experimental Procedures in SI). LC–MS intact mass analysis of deglycosylated immunoconjugates shows the degree of modification on full-length mAb, while LC–MS middle-up analysis of reduced immunoconjugates indicates the changes of each chain (Figure 1).

In detail, the immunoconjugate synthesized under kinetic control (KC) contained fewer conjugated species compared to that obtained from the one-step reaction (Figure 1, Intact). Calculated CAR were of 0.9 for the kinetically controlled synthesized immunoconjugate and 1.4 for the one-step synthesized. It is important to note that the increased modification rate of the one-step synthesized immunoconjugate did not impact the monoconjugated species (*T* + DOTA), but rather created a third family of conjugated species (*T* + 3DOTA). The ESI-MS spectra of the kinetically controlled reaction, acquired in middle-up modes, indicate *m*/*z* signals corresponding to the monoconjugated form (*T* + DOTA) suggesting the involvement of one lysine residue in the conjugation of both the heavy and light chain of the mAb (Figure 1, heavy and light chain). When the one-step reaction was applied, the mass spectrum acquired revealed *m*/*z* signals corresponding to mono- (*T* + DOTA) and biconjugated form (*T* + 2DOTA) in the heavy chain of mAb, indicating the involvement of more than one lysine residue within the same chain. These results suggested a more heterogeneous mixture of immunoconjugates when the reaction occurs under the same conditions but without a kinetic control.

**Figure 2:**
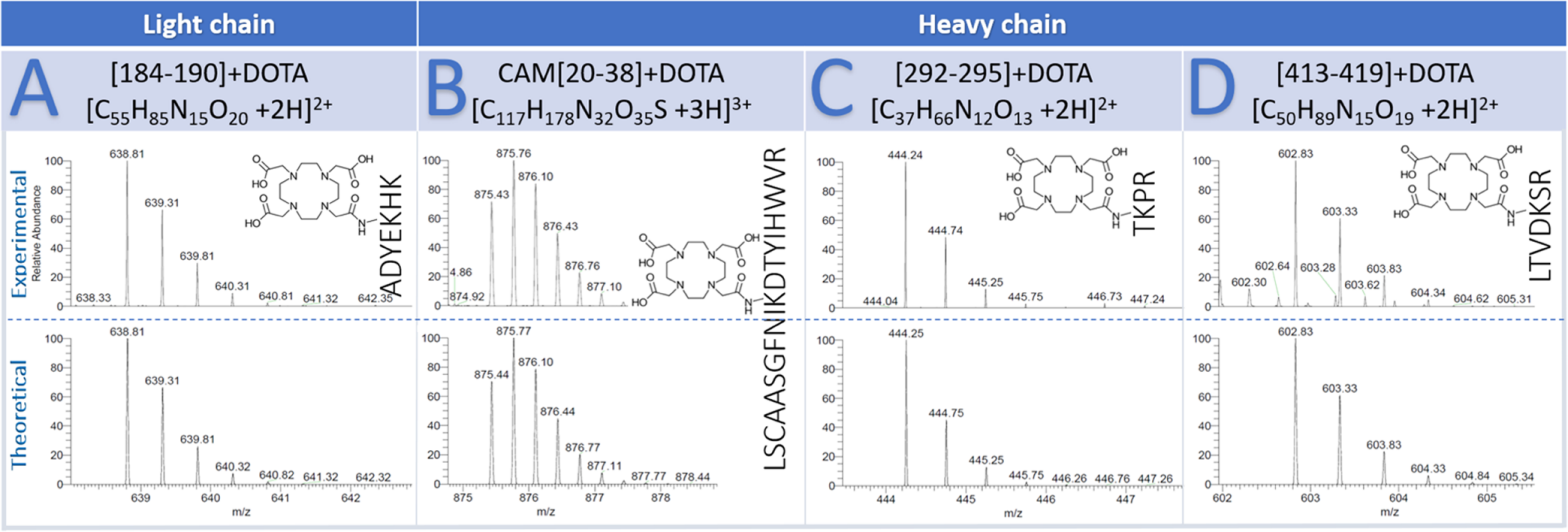
Comparison of experimental (top) and theoretical (bottom) spectra of DOTA-conjugated fragments: (A) *m*/*z* 638.81 (charge state +2) corresponding to the fragment [184–190] + DOTA (light chain); (B) *m*/*z* 875.43 (charge state +3) corresponding to the carbamidomethylated (CAM) fragment [20–38] + DOTA (heavy chain); (C) *m*/*z* 444.24 (charge state +2) corresponding to the fragment [292–295] + DOTA (heavy chain); (D) *m*/*z* 602.83 (charge state +2) corresponding to the fragment [413–419] + DOTA (heavy chain).

We resorted to proteolysis experiments to identify the lysine residues mainly involved in the reaction with DOTA-NHS and to verify if the conjugation reaction, under kinetic control, affected the CDRs of Trastuzumab. In particular, the conjugated protein was digested by a trypsin enzyme that selectively catalyzes the hydrolysis of peptide bonds at the C-terminal side of lysyl and arginyl residues. Usually, cleavage of the peptide bonds by a protease is rapid in an unfolded protein. Nevertheless, the steric hindrance of DOTA molecules bonded to lysine residues affects the accessibility to the amide bond, preventing its hydrolysis. Therefore, digested samples were analyzed by tandem mass spectrometry and high-resolution mass spectrometry techniques. The peptide mapping showed over 90% of the sequence covered (see the section “peptide mapping” in SI). DOTA-conjugated fragments were identified as partially digested peptides, containing one missed cleavage, since trypsin could not cut for the presence of DOTA or zero missed cleavage with the conjugated lysine followed by proline. Residues involved in bioconjugation corresponded to lysins located at the positions of the missed cleavage. The respective fragments (1 missed cleavage) DOTA-free were not found. Experimental spectra of fragments containing DOTA compared with its theoretical are shown in Figure 2. Residues mainly involved in bioconjugation were K188 in the light chain, whereas K30, K293, and K417 in the heavy chain. Traces of other conjugated residues were also found. Noteworthy, the four lysine residues mainly involved in the reaction with DOTA were localized in four different domains of Trastuzumab, namely, the VH, CH2, and CH3 domain of the heavy chain and CL domain of the light chain. This means that a regioselective bioconjugation occurred within each of these domains when the kinetic control was employed.

**Figure 3:**
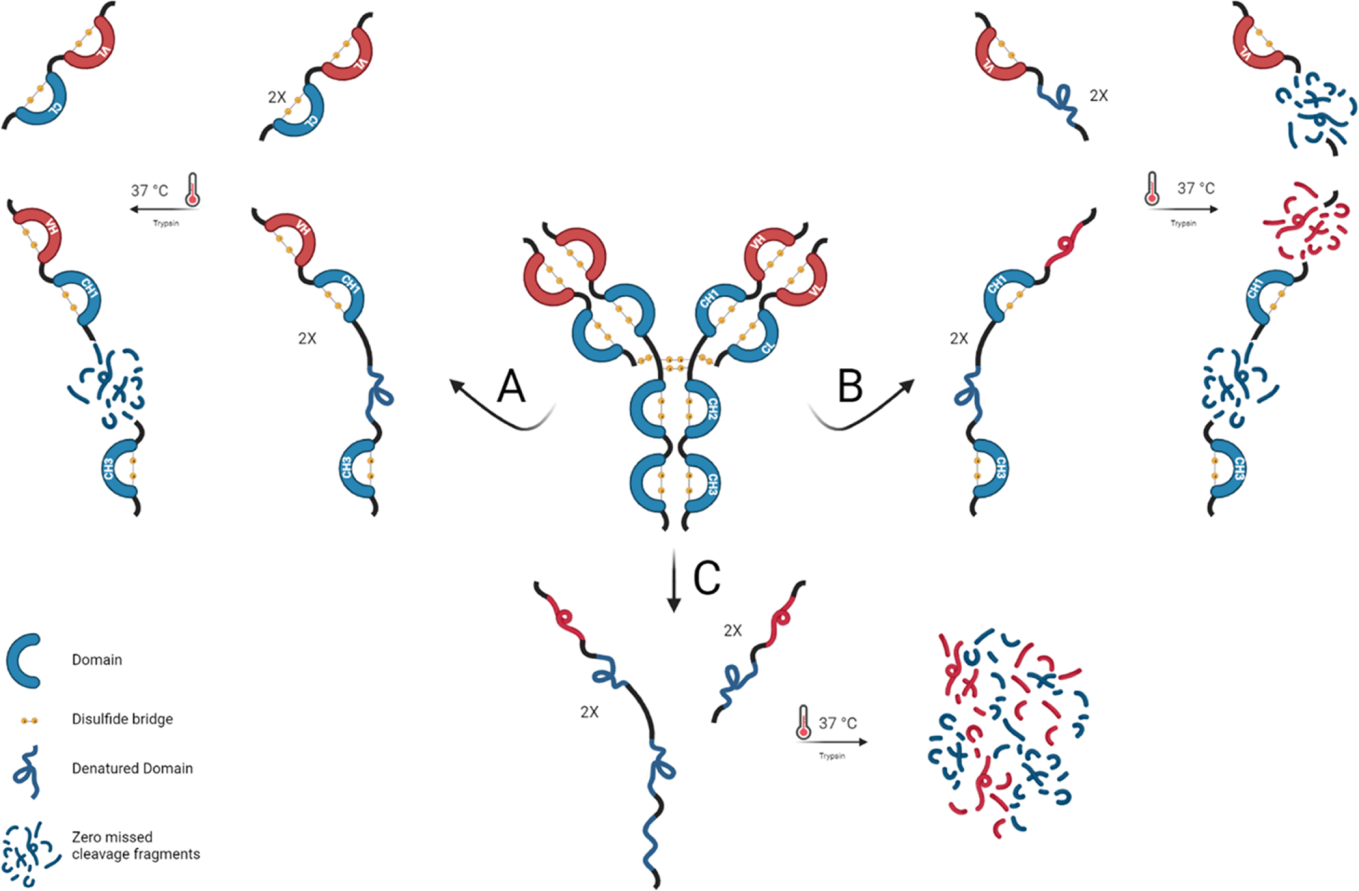
Schematic representation of the limited proteolysis protocol. Immunoconjugate, denatured at 68 °C during reduction and alkylation steps, followed three main pathways of unfolding. Pathway A: the only CH2 domain is denatured and fully digested, undigested Fab_HC_, CH3 domain fragments, and intact LC are generated; pathway B: CH2, VH, and CL domains are denatured and fully digested, generating CH3, CH1, and VL single-domain fragments; pathway C: the entire immunoconjugate is denatured and fully digested. Picture created with BioRender.com.

The percentage of DOTA bonded to the different lysine residues of Trastuzumab could be evaluated by comparing the peak areas of the partially digested fragments (conjugated) with those corresponding to the fully digested peptides. Unfortunately, this relative quantitative analysis is limited by the different lengths of compared peptide fragments and its different retention times during reversed-phase chromatography, which affect the ionization efficiency and significantly reduce the reproducibility of *m*/*z* signal intensity. The development of a limited proteolysis protocol capable of producing larger polypeptides should avoid changes in the retention time of the peptides after conjugation with DOTA, ensuring the coelution of these fragments. This enables the comparison, in the same spectrum, between the *m*/*z* signals corresponding to the conjugated and nonconjugated form of the peptide, limiting the problems associated with the variability of the *m*/*z* signal intensity. Considering the intradomain regioselectivity shown by the mass spectrometry studies of the fully digested immunoconjugate, the degree of modification of each residue had to match that of the entire corresponding domain. Thus, limited proteolysis experiments were designed to obtain polypeptides as large as entire domains, allowing for the relative quantification of conjugation. Therefore, we hypothesized that selective unfolding of some domains would subsequently allow trypsin to cut at the edges of the remaining folded. In detail, proteolysis was carried out using partial hot denaturation conditions (68 °C) during the reduction and alkylation steps (see the section “Experimental Procedures” in SI). In these conditions, specific domain denaturation can occur.^32,33^ As a matter of fact, the proteolytic pattern obtained after overnight trypsin digestion showed fragments derived from a mixture of differently unfolded immunoconjugates. In addition to the fragments derived by the fully digested immunoconjugates (Figure 3, pathway C), LC–MS analysis showed peptide fragments corresponding to whole undigested domains where intrachain disulfide bridges were not reduced, namely, Fab_HC_ (indicating the set of domains VH+CH1), CH3 and intact full-length LC (Figure 3, pathway A), and the domains VL and CH1 (Figure 3, pathway B).

**Figure 4:**
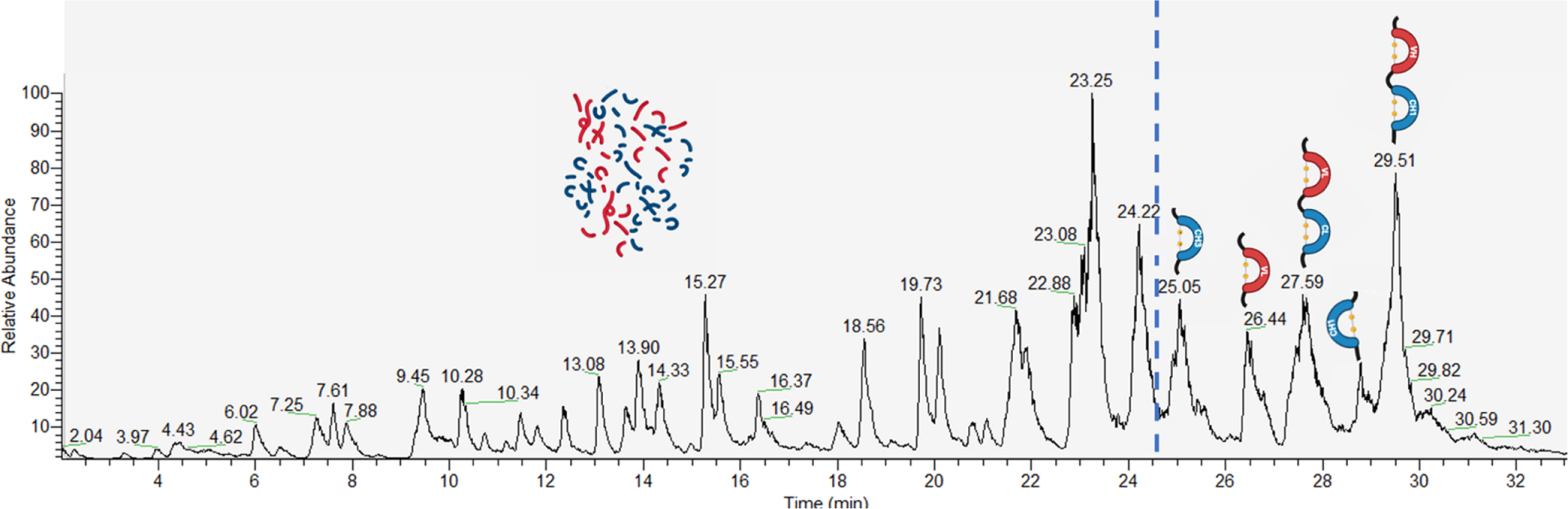
Domain mapping of the immunoconjugate synthesized under kinetic control.

The advantage of such a procedure was to assess the percentage of chelator conjugated to each domain, since unmodified and modified large polypeptides chromatographically coelute, without the support of quantitative investigation techniques based on isotopic chemical labeling. Domain and peptide fragments identification was performed with assistance of an in silico digestion on mMass software, comparing simulated with experimentally obtained spectra. Regions of chromatographic interest are indicated in Figure 4.

**Figure 5:**
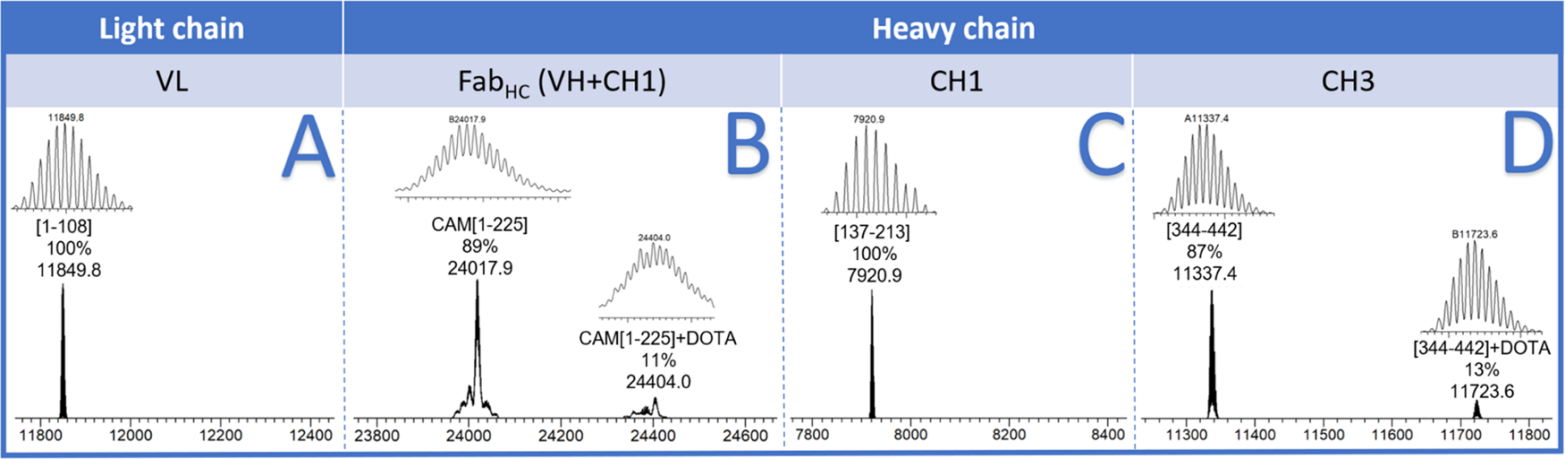
Deconvoluted spectra of peaks eluting from 25 min onward: (A) fragment [1–108] of the light chain corresponding to the VL domain, no presence of DOTA-conjugated species was revealed; (B) fragment [1–225] carbamidomethylated (CAM) at C223 of the heavy chain corresponding to domain Fab_HC_, 11% of its DOTA-conjugated was revealed; (C) oxidized fragment [137–213] of the heavy chain corresponding to the CH1 domain, no presence of DOTA-conjugated species was revealed; (D) fragment [344–442] of the heavy chain corresponding to domain CH3, 13% of its DOTA-conjugated was revealed.

The chromatogram (Figure 4) could be divided into two main regions, the first ranging from 2 to 25 min containing fragments derived from the fully digested immunoconjugate, while the second from 25 min onward corresponds to undigested domains. In detail, peaks eluting at RT 25.05 holds the fragment [344–442] corresponding to the undigested CH3 domain; RT 26.44 the LC fragment [1–108] corresponding to the domain VL; RT 27.59 to the fully undigested light chain; RT 28.90 the oxidized HC fragment [137–213] corresponding to CH1 domain; and RT 29.51 the HC fragment [1–225]. The ESI-MS spectra extracted from each of these chromatographic peaks showed the coelution of unmodified and conjugated forms (Figure 5), allowing us to assess the percentage of chelator bound to each domain (Table 1).

**Table 1:** Percentage of DOTA Bonded to Each mAb Domain

domain	measured fragment	% DOTA
VH		11^a^
CH1	[137–213]	0
CH2		12.5^b^
CH3	[344–442]	13
VL	[1–108]	0
CL		6^c^

a Value calculated using the formula (VH + DOTA) = [(Fab_HC_ + DOTA) – (CH1 + DOTA)].

b Value calculated using the formula (CH2 + DOTA) = {(HC + DOTA) – [(Fab_HC_ + DOTA) + (CH3 + DOTA)]}.

c Value calculated using the formula (CL + DOTA) = [(LC + DOTA) – (VL + DOTA)].

Conjugated forms of Fab_HC_ and CH3 domains were quantified, whereas no presence of conjugation was detected in the domains CH1 and VL, according to the results obtained by peptide mapping of the fully digested immunoconjugate. Therefore, the cross analysis of the data obtained from domain mapping and middle-up analysis of reduced chains allowed for calculations of the percentage of chelator linked to each mAb domain. Resulting data are summarized in Table 1.

**Figure 6:**
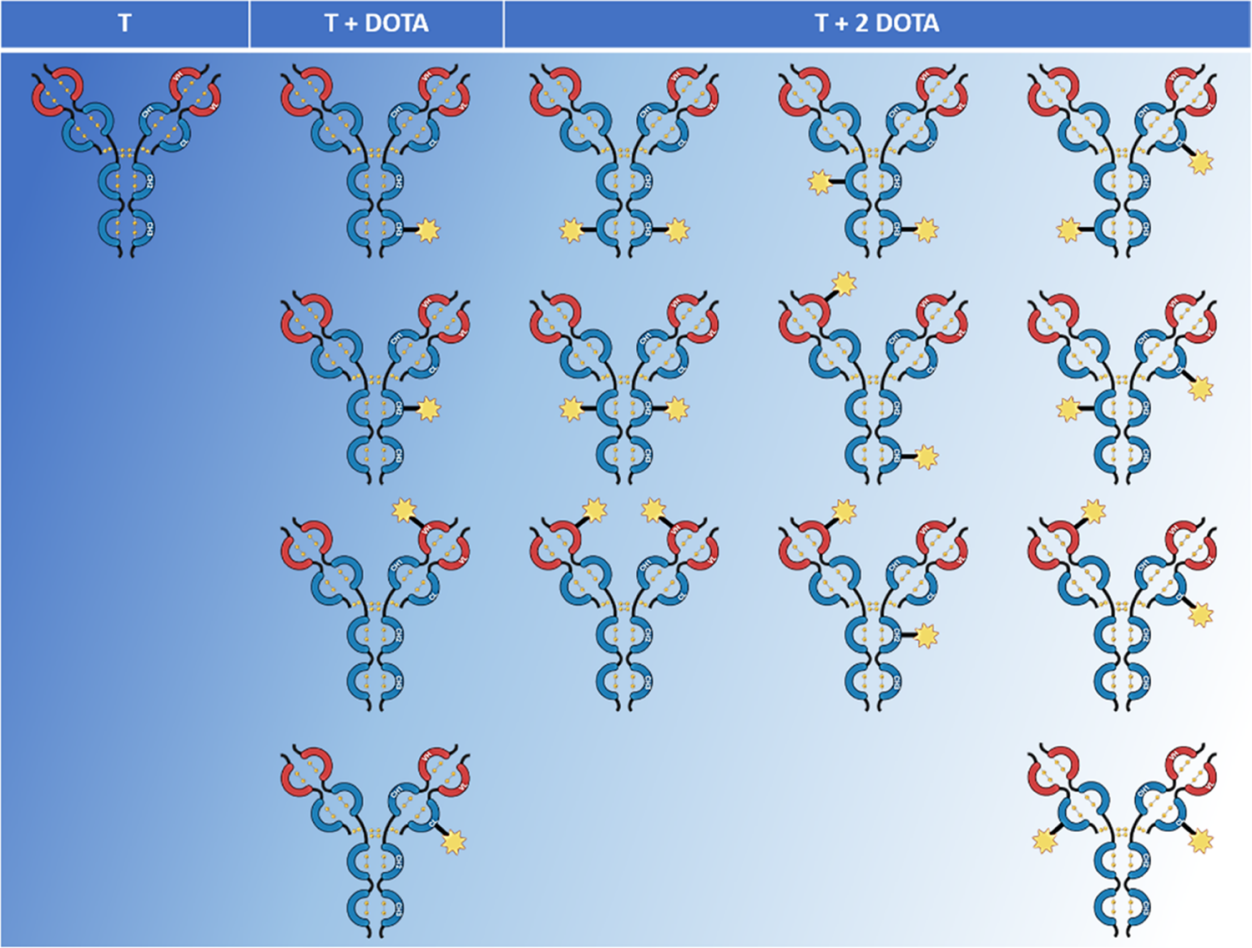
Representation of mixture composition: blue color indicates the most intense species, white color the less. Created with BioRender.com.

Concerning the bioconjugation in the CDR_s_, Trastuzumab contains only two lysine residues in those regions, both in the heavy chain at K30 and K65. The K30 residue contained in CDR-H1, resulted in one of the most modified K residues after bioconjugation. Although it is known the region mainly responsible for mAb-antigen interaction is the CDR-H3,^34^ binding studies are currently designed by our group to evaluate how much this modification compromises the affinity of Trastuzumab for the sub domain IV of HER2. However, from a chemical point of view, we were able to determine the number of species containing this modified K residue and to calculate its relative percentage in the immunoconjugate mixture thanks to the developed domain mapping. Indeed, analysis of quantitative data obtained from domain mapping, middle-up, and intact mass analysis provided a molecular formulation of the immunoconjugate mixture (Figure 6). Since we knew the relative percentage of the three groups of species (*T*, *T* + DOTA, and *T* + 2DOTA) and that only one DOTA was conjugated to each chain (Figure 1), assuming each conjugated domain having a single lysine residue DOTA-modified, due to the intradomain regioselectivity shown above, from domain mapping results it was possible to calculate the percentage of each species in the mixture of immunoconjugates according to the generic formula a:
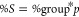
aWhere according to the group of which the species is part, *p* can be
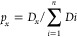

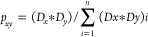


***S*** indicates generic conjugated species.

***x*** and ***y*** indicate the conjugated domain of the species (VH, CH1, CH2, etc.). *xx* and *yy* combinations are considered.

**% group** (*T* + DOTA or *T* + 2DOTA) values are measured in intact mass analysis.

***p*** indicates the weight of species *x* or *xy* within its group.

***D*** indicates the percentage of the conjugated domain derived from the domain mapping analysis.

Developing eq a for generic species *x* within the group *T* + DOTA and for the generic species *xy* within the group *T* + 2DOTA, eqs 1 and 2 are respectively obtained:
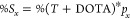
1
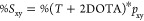
2

As we hypothesized a mixture restricted to 15 different species was obtained using a kinetic control, where up to 10^6^ species are statistically possible when one-step conjugations are employed, as in the case of drug-antibody conjugates having a drug-antibody ratio (DAR) value ranging from 2 to 4.^35^Table 2 shows the abundance of each species in the mixture, calculated using formula a.

**Table 2:** Percentage Composition of the Immunoconjugate Mixture

*S*	%
*T*	35
*S*_CH3_	13.46
*S*_CH2_	12.94
*S*_VH_	11.39
*S*_CL_	6.21
*S*_CH3-CH3_	3.10
*S*_CH2-CH3_	2.98
*S*_CH2-CH2_	2.87
*S*_VH-CH3_	2.62
*S*_VH-CH2_	2.52
*S*_VH-VH_	2.22
*S*_CH3-CL_	1.43
*S*_CH2-CL_	1.38
*S*_VH-CL_	1.21
*S*_CL-CL_	0.66

The most abundant species in the mixture resulted in the naked Trastuzumab, with the species *T* + 1 DOTA having a relative abundance in comparison to species *T* + 2DOTA ranging from 4 to more than 20-fold. The species containing the K30 residue DOTA-modified, i.e., VH-modified, were five and accounted for 19.96% of the total mixture. How much these VH-modified species influence the affinity for the antigen will be studied through binding radio-assays.

During the past decade, research in radioimmunoconjugates moved toward a lower CAR, in order that a mAb molecule carried with it fewer radionuclides. Beyond limiting the degree of modification to preserve mAb immunoreactivity, reduced CAR means more radiolabeled probes for the same quantity of radioactivity or even better reduced radioactive dose to patients to obtain a tumor to background ratio similar to that of radioimmunoconjugates with higher CAR. In the current study, we demonstrate that it is possible to synthesize immunoconjugates having unitary CAR achieving an intradomain regioselectivity, using commercially available reactants and working on mAbs in their native form, through a kinetically controlled bioconjugation. Although under these reaction conditions a complete bioconjugation of the mAb is not achievable, the synthesized mixture should ensure improved affinity for the antigen and lower radioactive dose to patients in comparison to immunoconjugates one-step synthesized. Lower radioactive dose would mean reduced radiotoxicity, paving the way for the approval of radioimmunoconjugates by regulatory agencies. DOTA-Trastuzumab derivatives were synthesized with theragnostic aims, owing to the ability of DOTA to complex several radionuclides for imaging (In-111, Ga-68, Sc-44, Cu-64) and therapy (Lu-177, Y-90, Sc-47, Ac-225). Future imaging procedures with DOTA-Trastuzumab compared with FDG-PET should provide a measurement of tissue penetration and tumor uptake of Trastuzumab, giving the oncologist a more accurate mapping of the biologic treatment. On the other hand, the physician will be able to plan a Trastuzumab-based radionuclide therapy thanks to the dosimetry data obtained from DOTA-Trastuzumab imaging.

Notably, the developed domain mapping MS-workflow resulted in a fundamental tool to characterize the mixture. It allowed for recognition of whole domains working in a borderline middle-up/bottom-up approach, allowing us to evaluate the percentage of modification for each of them and to calculate the percentage composition of the mixture. The knowledge of the weight of each species within a mixture could have dramatic implications in the development of immunoconjugates. First, future correlation studies between mixture compositions and higher order structure studies (HOSs) may result in predicting secondary, tertiary, and quaternary arrangements for immunoconjugates. Second, it could provide key information to streamline the extensive in vitro studies, such as affinity and immunoreactivity essays, currently necessary to demonstrate that some features of the mAb have not been lost during bioconjugation. We believe this named “domain mapping” could also be useful to broader application fields. Close to what is described in this study, it could be employed in routine analytical tests and stability studies of biopharmaceuticals. Moving away a little further, this selective domain unfolding could result interesting for other proteomics studies where proteolysis plays a key role, e.g., protein–protein interaction and protein structure investigation, especially in protein cross-linking and chemical labeling. Lastly, looking from a noninvestigative perspective, fragments obtained from this selective proteolysis could be purified and employed as future vectors for radionuclides or drugs. This perspective is particularly fascinating to us, especially for the generation of a new theranostic agent based on the single-domain VL of Trastuzumab. Currently, single-domain antibodies (sdAb) or nanobodies are obtained immunizing camelids and sharks with the antigen of interest; then, the nanobody is expressed in microorganisms, mammalian cells, and plants.^36^ Even if minimal, humanization is a crucial step to reduce their immunogenicity.^37^ However, careful consideration must be made regarding the solubility of the VL domain of Trastuzumab. Note that, whatever the purpose for which the domain mapping will be used, limitations mainly arising from the experimental procedure should be considered. Especially, overalkylation and oxidation of some fragments can occur at operating temperatures over time; thus, the method should be tuned to achieve the desired purpose.

## Conclusions

In the present study, we demonstrate that it is possible to synthesize a mixture of immunoconjugates restricted to few species using a kinetically controlled reaction where an intradomain regioselectivity is achieved, this obtained using not tailored site-specific reactant, but commercially available. Owing to its unitary CAR, the DOTA-Trastuzumab derivative synthesized under kinetic control should guarantee reduced radiotoxicity in comparison to traditionally synthesized radioimmunoconjugates. Moreover, the presence of the DOTA chelator allows for theragnostic applications. Although the results of bioconjugation are limited to the case study, we believe the kinetically controlled approach can be extended to other IgG1 molecules, owing to the large number of sequence homologies shared within this immunoglobulin subclass. Immunoconjugate species within the mixture were identified, and their percentages were calculated by developing a new proteolysis protocol named domain mapping (patent application IT2024000001524). The latter coupled with LC–MS analysis allowed for quantification of modified domains. This domain mapping might be useful for broader omics studies, where proteolysis plays a key role and as a preparation method for single-domain antibodies. In conclusion, the coupling of synthesis under kinetic control with its monitoring using domain mapping could provide a model to obtain immunoconjugates which ensure a pharmaceutical quality, nowadays not achievable with traditional bioconjugation employing activated esters.

## Supporting Information Available

Experimental procedures and peptide mapping (PDF)

## Abbreviations

mAb: monoclonal antibody
CAR: chelator-antibody ratio
T: Trastuzumab
HER2: human epidermal growth factor receptor 2
KC: kinetically controlled
RP: reverse phase
MS: mass spectrometry
CAM: carbamidomethylated
Fab_HC_: fragment antigen binding limited to the heavy chain (corresponding to the set of VH + CH1 domains)
