# Supplementary material for: A Kinetically Controlled Bioconjugation Method for the Synthesis of Radioimmunoconjugates and the Development of a Domain Mapping MS-Workflow for Its Characterization
Source: Bioconjug Chem. 2024 Feb 17;35(3):324–32. doi: 10.1021/acs.bioconjchem.3c00519 (PMC10961728; doi:10.1021/acs.bioconjchem.3c00519)
Supplement: Supplementary file 1 — bc3c00519_si_001.pdf [file bc3c00519_si_001.pdf]

Supporting information

A kinetically controlled bioconjugation method for the synthesis of radioimmunoconjugates and the development of a domain mapping MS-workflow for its characterization.

Marco A. Pometti, [*, ⊥, †] Giuseppe Di Natale, [‡] Giancarlo Geremia, [⊥, §] Nileshgiri Gauswami, [⊥, §] Gianni Garufi, [⊥, §] Giuseppina Ricciardi, [⊥, †] Marcella Sciortino, [⊥, †] Fabrizio Scopelliti, [⊥] Giorgio Russo, [‖] Massimo Ippolito [⊥]

[⊥] Nuclear medicine department, Cannizzaro Hospital, Via Messina 829, 95126, Catania, IT

[†] FORA S.p.A., Via Alfred Bernhard Nobel 11/a, 43122, Parma, IT

[‡] CNR-Istituto di Cristallografia, Via Paolo Gaifami 18, 95126 Catania, IT

[§] Parco scientifico e tecnologico della Sicilia S.C.P.A., Stradale Vincenzo Lancia 57, 95121 Catania, IT

IBFM-CNR Institute of molecular Bioimaging and Physiology, Contrada Pietra Pollastra, 90015, Cefalù, IT

*Corresponding Author

Tel: +39 3493470527; E-mail: marco.pometti@gmail.com

https://orcid.org/0009-0004-6399-6076

EXPERIMENTAL PROCEDURES

Materials. Trastuzumab 150 mg (Trazimera, pfizer) was kindly provided by the Pharmacy department of fondazione istituto G. Giglio Cefalù. DOTA-NHS (C084) was purchased from chematech-mdt; Trypsin platinum mass spectrometry grade from Promega; Endosafe Tubes 13x100 mm (T300-50) from Charles River; Intact mAb check standard (186006552) from Waters. All the other reagents and consumables were purchased from Sigma-Aldrich: Disposable PD 10 Desalting Columns (GE17-0851-01); Sodium phospate dibasic heptahydrate (431478-50G); Sodium phosphate monobasic monohydrate (71507-250G); Sodium Carbonate (S7795-500G); Sodium hydrogen carbonate (1.37013.1000); TRIS HCl (1083150100); Tris Base (93362-500G); PNG-ase F from Elizabethkingia miricola (G5166-50UN); CaCl2 1M (21115-100ML); DTT (3870-25GM); Iodoacetamide (Cytiva, GERPN6302); Water for chromatography (LC-MS Grade) LiChrosolv (1153334000); ACETONITRILE hypergrade for LC-MS LiChrosolv" (1000294000); Formic acid LC-MS (5.33002.0050).

Preliminary steps. 2,5 mL of Trastuzumab 21mg/mL were purified and carried in phosphate buffer 0,05 M pH 7,2, using a gravity protocol on PD-10 Desalting columns, obtaining a new volume of 3,5 mL and a new concentration of 13,5 mg/mL.

Intact mAb Mass Check Standard (Waters) was used as a reference sample for LTQ-XL instrument tuning and calibration check for intact mass analysis.

Kinetically controlled bioconjugation. A 500 µL Hamilton syringe, driven by the infusion pump of the mass spectrometer LTQ-XL and filled with DOTA-NHS 685 µM solution, ran to 2uL/min (1,37 nmol/min) adding it to 1,5 mL of Trastuzumab 13,5 mg/mL (137 nmol) in phosphate buffer pH 7,2 contained in a glass tube (figure 1). The syringe was refilled every 25 minutes with a fresh reconstituted DOTA-NHS 685 µM solution, aiming to keep its concentration constant. Reaction was monitored every 100 minutes. In particular, 50 µg of the immunoconjugate produced, were reduced with 1,25 mM DTT solution for 30 minutes at 37°C. Afterwards, 0,1 µg of reduced sample was injected into LC-MS. An RP-HPLC 5%/min gradient method (modifier CH_3CN:IPA 30:70; 0,1% formic acid; flow 450uL/min) was employed using DIONEX ultimate 3000 µHPLC equipped with Phenomenex Biozen 2,6 µm widepore C4 (150x2,1mm) and coupled with Thermo LTQ-XL mass spectrometer. The instrument was operated applying the following ESI source ionization parameters: sheath gas 45 arb, aux gas 30 arb, I spray voltage 4 KV, capillary temperature 290°C, capillary voltage 28V, tube lens 220V. ESI-spectra were acquired in the range 1000-2000 m/z using AGC target 3e4 with maximum IT 200ms. MagTran.exe software was used for deconvolution. Once the bioconjugation achieved the desired degree, the resulting immunoconjugate was purified and desalted through fractional separation on PD-10 desalting columns using a gravity protocol, collected in 7 fractions containing 0,5 mL each one and stored at 4°C. The different enrichment of fractions was assessed through HPLC-UV.

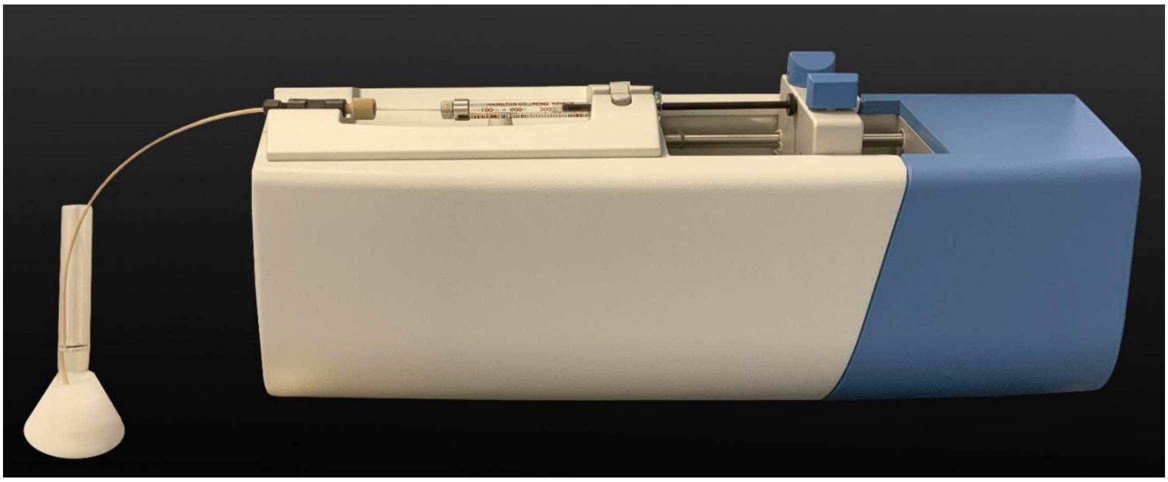

Figure 1. DOTA-NHS added to a vial containing Trastuzumab through a Hamilton syringe running at 2 uL/min driven by the infusion pump of the mass spectrometer LTQ-XL.

One-step bioconjugation. 500µL of 1,37 mM DOTA-NHS solution (685 nmol) were added to 1,5 mL of Trastuzumab 13,5 mg/mL (137 nmol) contained in a glass tube.

The bioconjugation was carried out for 2 hours at room temperature. The resulting immunoconjugate was purified, desalted and LC-MS characterized using the same protocols reported for the kinetically controlled bioconjugation.

Intact mass analysis. 300 µg of each immunoconjugate were deglycosylated by digestion with 1 unit of PNGase F at 37°C overnight in Tris buffer pH 7,6. LC-MS analysis of deglycosylated immunoconjugates were conducted in denaturing conditions using 15%/min gradient method (modifier CH3CN:IPA 30:70 0,1% formic acid; flow 450uL/min), using DIONEX ultimate 3000 µHPLC equipped with Phenomenex Biozen 2,6 µm widepore C4 (150x2,1mm) and coupled with Thermo LTQ-XL mass spectrometer. The instrument was operated applying the following ESI source ionization parameters:sheath gas 45 arb, aux gas 30 arb, I spray voltage 4 KV, capillary temperature 290°C, capillary voltage 28V, tube lens 220V. Spectra were acquired in the range 2300-3700 *m/z* using AGC target 3e4 with maximum IT 200ms. MagTran.exe software was used for spectra deconvolution.

Middle-up analysis. Immunoconjugates were reduced using DTT 1,25 mM and subsequently digested overnight with 1 unit of PNGase F to better assess the degree of modification on the heavy chain. LC-MS analyses were conducted in denaturing conditions using a 5%/min gradient method (modifier CH3CN:IPA 30:70 0,1% formic acid; flow 450uL/min). Instruments and ESI ionization methods were the same used in intact mode. ESI-spectra were acquired in the range 1000-2000 m/z using AGC target 3e4 with maximum IT 200ms. MagTran.exe software was used for deconvolution.

Domain mapping MS-workflow. 300 µg of immunoconjugate synthesized through the kinetically controlled method was reduced with DTT 5 mM in tris buffer pH 7,6 for 30 minutes at 68°C. Iodoacetamide was added to the reaction mixture to reach a final concentration of 25 mM and reacted for 30 minutes at 68°C. Afterwards, 30 ug of reduced and alkylated immunoconjugate were digested overnight at 37°C with trypsin platinum mass spectrometry grade (Promega), enzyme:antibody ratio 1:20, in tris buffer pH 7,6 at 37°C. Digestion was monitored every hour for 6 hours and overnight through RP-LC-MS using DIONEX ultimate 3000 µHPLC equipped with Phenomenex Biozen C4 and coupled with Thermo LTQ-XL mass spectrometer. Analyses were conducted using a 1%/min gradient method (modifier CH3CN 0,1% formic acid; flow 300µL/min). ESI source ionization parameters were sheath gas 35 arb, aux gas 10 arb, I spray voltage 4 KV, capillary temperature 275°C, capillary voltage 28V, tube lens 220V. Spectra were acquired in the range 500-2000 *m/z* using AGC target 3e4 with maximum IT 200ms. Confirmations of mass measurements were carried out on the overnight digested through nLC-nESI HRMS using a Dionex ultimate 3000 equipped with Easy-spray column Accucore C4 (2,6 µm, 75 µm x 150 mm) and coupled with Q-Exactive plus mass spectrometer (Thermo Fisher Scientific). Analyses were conducted using a 1%/min gradient method (modifier CH3CN 0,1% formic acid; flow 0,3 µL/min). nESI parameters were spray voltage 2,5 kV without sheath gas. Spectra were acquired in the range 300-2000 *m/z* using the following parameters: 70 K resolution (@200 *m/z*), AGC target 3e6, maximum IT 200ms.

Peptide and domain identification. mMass software was employed for in silico digestion and fragments identification. The following parameters were used:

Light chain sequence modification (all variables): Carbamidomethyl (+57,0215 Da) at all C; DOTA (+386,1801 Da) at all K and N-terminus.

Heavy chain sequence modification (all variable): G0F (+1444,5339 Da), G1F (+1606,5867 Da), G2F (+1768,6395 Da) at N300; Carbamidomethyl (+57,0215 Da) at all C; DOTA (+386,1801 Da) at all K and N-terminus.

Digestion parameters: Enzyme: Trypsin; Missed cleavage: from 0 to 30; Mass range 100-30000 Da.

Advice. Overalkylation can occur at 68°C, take this into account during peptide identification. No unexpected or unusually high safety hazards were encountered during experimental procedures.

PEPTIDE MAPPING

Light Chain

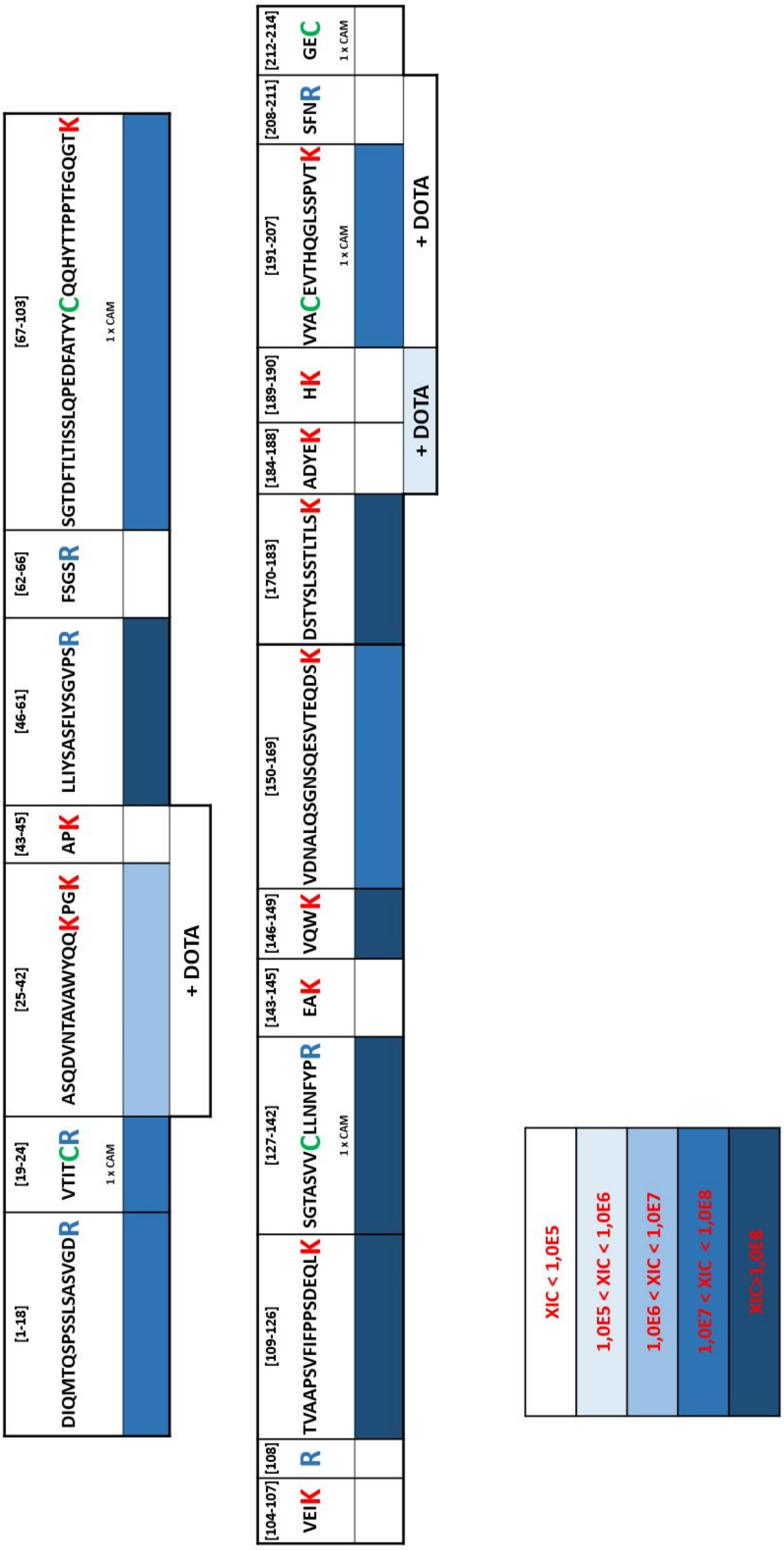

Figure 2A. Peptide mapping of the light chain

Heavy Chain

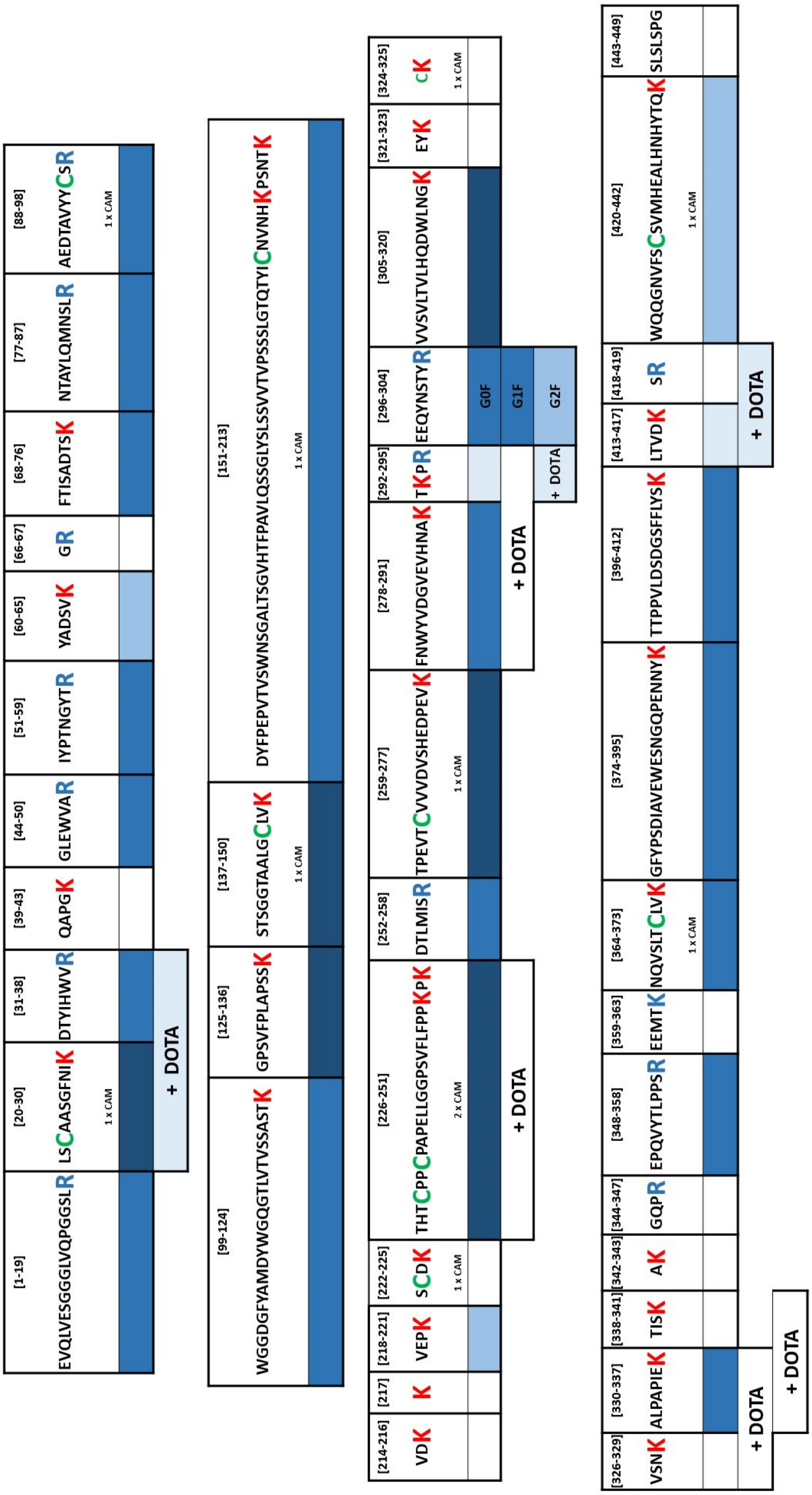

Figure 2B. Peptide mapping of the heavy chain
